# Epidermal Growth Factor Receptor (EGFR)-Mutated Non-Small-Cell Lung Cancer (NSCLC)

**DOI:** 10.3390/ph13100273

**Published:** 2020-09-25

**Authors:** Connor O’Leary, Harry Gasper, Katherine B. Sahin, Ming Tang, Arutha Kulasinghe, Mark N. Adams, Derek J. Richard, Ken J. O’Byrne

**Affiliations:** 1 Princess Alexandra Hospital, Brisbane 4000, Australia; harry.gasper@health.qld.gov.au (H.G.); k.obyrne@qut.edu.au (K.J.O.); 2 School of Biomedical Sciences, Faculty of Health, Queensland University of Technology, Brisbane 4000, Australia; Katherine.sahin@hdr.qut.edu.au (K.B.S.); m21.tang@qut.edu.au (M.T.); arutha.kulasinghe@qut.edu.au (A.K.); mn.adams@qut.edu.au (M.N.A.); derek.richard@qut.edu.au (D.J.R.); 3 Cancer and Ageing Research Program, Translational Research Institute, Brisbane 4000, Australia

**Keywords:** non-small-cell lung cancer, epidermal growth factor receptor, EGFR, tyrosine kinase inhibitors, circulating tumor cells

## Abstract

Epidermal growth factor receptor (EGFR) mutations are the most common oncogenic drivers in non-small-cell lung cancer (NSCLC). Significant developments have taken place which highlight the differences in tumor biology that exist between the mutant and wild-type subtypes of NSCLC. Patients with advanced EGFR-mutant NSCLC have a variety of EGFR-targeting agents available proven to treat their disease. This has led to superior patient outcomes when used as a monotherapy over traditional cytotoxic systemic therapy. Attempts at combining EGFR agents with other anticancer systemic treatment options, such as chemotherapy, antiangiogenic agents, and immunotherapy, have shown varied outcomes. Currently, no specific combination stands out to cause a shift away from the use of single-agent EGFR inhibitors in the first-line setting. Similarly, adjuvant EGFR inhibitors, are yet to significantly add to patient overall survival if used at earlier timepoints in the disease course. Liquid biopsy is an evolving technology with potential promise of being incorporated into the management paradigm of this disease. Data are emerging to suggest that this technique may be capable of identifying early resistance mechanisms and consequential disease progression on the basis of the analysis of blood-based circulating tumor cells.

## 1. Introduction

Treatment of lung cancer, particularly with the adenocarcinoma subtype, has evolved significantly in recent years. A notable change is the analysis of patient tumors for the presence of oncogenic drivers [1]. With ongoing research, the number of actionable mutations is steadily increasing, offering a more personalized approach to the management of patients with lung cancer [2,3]. 

Epidermal growth factor receptor (EGFR)-mutated lung cancer occurs in 15–20% of patients with adenocarcinoma and is most commonly associated with nonsmokers and those of Asian ethnicity. Targeted EGFR treatment offers superior outcomes and toxicity profiles compared to traditional cytotoxic-based treatments in those with advanced disease. In this article, we outline the underlying mechanisms of action of the various EGFR tyrosine kinase inhibitors (TKIs), clinical data, and the evolving role of liquid biopsy in this disease. 

## 2. Pathophysiology and EGFR TKI Mechanisms of Action

EGFR is a receptor tyrosine kinase (RTK) consisting of an amino-terminal extracellular domain, a single membrane-spanning domain, and a cytoplasmic domain. Activation of the wild-type receptor is mediated by the binding of one of six endogenous ligands, including epidermal growth factor (EGF), to the extracellular domain to induce a conformational change that promotes EGFR family member homo- or heterodimerization. The cytoplasmic region of EGFR contains a catalytic tyrosine kinase domain and sites of docking for molecular interactions with downstream signaling effector proteins. Dimerization of EGFR promotes autophosphorylation on tyrosine residues within the intracellular domain and stimulates the Ras/Raf/MAPK and PI3K/Akt/mTOR signaling pathways that orchestrate a range of cellular processes such as migration, proliferation, and cell survival.

EGFR mutations are located in the catalytic tyrosine kinase domain of EGFR as a small in-frame deletion within exon 19 (E19del) or as a leucine-to-arginine point mutation at codon 858 (L858R) within exon 21 [4]. The resulting effect is that the EGF receptor is constitutively activated [5]. The L858R mutations act by stabilizing the activation loop of the receptor and increasing the duration of ligand-dependent activation of EGFR [6]. The E19del mutations work in much the same way. Deletion of exon 19 results in a conformational shift in the EGFR helical axis, resulting in a narrowed ATP-binding cleft and increased ligand-dependent activation of the EGFR [6]. These first-generation TKIs work against the activating EGFR mutations L858R and E19del by inhibiting EGF-induced autophosphorylation of the EGF receptor [7,8].

Further investigation identified an acquired threonine-to-methionine mutation at codon 790 (T790M) within exon 20 in tumors of patients who developed resistance to first-generation EGFR TKIs [9,10,11]. Other additional resistance mechanisms to first-generation TKIs were also reported, such as small in-frame insertions in exon 20 of the kinase domains of EGFR and human epidermal growth factor receptor (HER2/ErbB2) [12,13]. 

A more broad-spectrum inhibitor against receptor tyrosine kinases with these acquired and primary resistance mechanisms is required. Afatinib, a pan-ErbB family inhibitor, presented as an attractive therapeutic option, given its ability to block EGFR, HER2, and HER4 (ErbB4), has wide-spectrum preclinical activity against EGFR mutations [14,15]. 

Afatinib covalently binds to cysteine 797 of the EGFR and cysteines 805 and 803 of HER2 and HER4, respectively. In doing so, the second-generation TKI irreversibly inhibits the TKI activity of these receptors. As a result, auto- and transphosphorylation within the ErbB dimers is decreased, thus reducing the activity of downstream signaling pathways [16]. 

However, clinical trials comparing the treatment of afatinib alone or in combination with chemotherapy to chemotherapy alone found that they had limited added therapeutic benefit relative to first-generation EGFR TKIs due to the adverse effects they present such as stomatitis, paronychia, anorexia, diarrhea, and rash. This also included dose-limiting toxicity via inhibition of wild-type EGFRs in addition to inhibiting mutated EGFRs [17,18,19]. 

A 2018 study looking at the worldwide frequency of commonly detected EGFR mutations identified that the T790M mutation occurs in 0.7% of all NSCLC cases [20]. Furthermore, the T790M mutation accounts for approximately half of all cases with resistance to gefitinib and erlotinib [21,22]. The T790M mutation sits within the ATP-binding pocket of the catalytic tyrosine kinase domain of the EGFR. The T790M mutation affects the binding kinetics of the TKI to EGFR by enhancing the affinity of the receptor for ATP and reducing the potency of competitive EGFR TKIs [23]. Third-generation TKIs, such as osimertinib, were developed to selectively and irreversibly target T790M mutations [14]. Similar to second-generation TKIs, they function within the catalytic domain at the ATP-binding pocket to displace ATP. However, their mode of action is different to second-generation TKIs in that osimertinib binds to the EGFR irreversibly by targeting and covalently binding to the cysteine 797 residue (C797) in the ATP-binding pocket [14]. 

Despite the success of third-generation TKIs, mutations mediating resistance to third-generation TKIs have emerged alongside the T790M mutation, such as the cysteine-to-serine point mutation at codon 797 (C797S) in exon 20 [24]. The C797S mutation is thought to induce resistance to TKIs by impairing the covalent binding of these TKIs to the EGFR [25]. Other identified tertiary point mutations include L798I, L718Q, L692V, E709K, L844V, and G796D [11,24,26,27,28]. 

It is clear that further study is required to explore the mutational heterogeneity of the EGFR with particular focus on the prevalence of emerging mutations with the known sensitizing (E19del and L858R) and resistant (T790M) mutations. The broad mutational landscape of EGFR indicates the need for combination therapies that can simultaneously inhibit or prevent the emergence of multiple resistance mechanisms.

## 3. EGFR Monotherapy as Palliative Therapy in Advanced Disease

### 3.1. First-Generation EGFR Tyrosine Kinase Inhibitors

Two first-generation EGFR tyrosine kinase inhibitors (TKI) inhibitors, erlotinib and gefitinib, are currently available for patients with advanced/incurable EGFR exon 19 deletion and exon 21, L858R, mutated NSCLC. These drugs are well established in clinical practice and widely used in the first-line treatment of patients with EGFR-mutated NSCLC.

Erlotinib was evaluated in three first-line phase III studies in both Asian and Caucasian populations (Table 1). The OPTIMAL study performed by a Chinese group evaluated erlotinib against first-line platinum-based chemotherapy. Median progression-free survival (PFS) was significantly longer in the erlotinib arm: 13.1 versus 4.6 months, hazard ratio (HR) 0.16, *p* < 0.0001 [29]. Erlotinib was subsequently evaluated in a similar setting by a European group in the EURTAC trial. This trial also found an improvement of median PFS in the experimental arm: 9.7 versus 5.2 months, HR 0.37, *p* < 0.0001 [30]. Another phase III Asian study, ENSURE, compared erlotinib to first-line cisplatin and gemcitabine. A significantly improved median PFS was observed: 11 versus 5.5 months, HR 0.34, *p* < 0.0001 [31]. 

Gefitinib was studied in a number of first-line trials (Table 1). A Japanese group, WJTOG3405, reported a median PFS of 9.2 versus 6.3 months, HR 0.489, *p* <0.0001, when gefitinib was compared to first-line cisplatin and docetaxel. [32]. Another Japanese trial evaluated gefitinib compared to carboplatin and paclitaxel combination chemotherapy. Again, an improvement in median PFS was observed in the gefitinib group: 10.8 versus 5.4 months, HR 0.30, *p* < 0.001. Post hoc analysis of the IRESSA Pan-Asia Study (IPASS) of patients with a confirmed EGFR mutation confirmed superiority of gefitinib in this cohort: PFS 9.5 versus 6.3 months, HR 0.48, *p* < 0.001 [33]. 

### 3.2. Second-Generation EGFR Tyrosine Kinase Inhibitors

Second-generation EGFR TKIs work by irreversibly binding the tyrosine kinase domain of EGFR along with other members of the ErbB family, thereby inhibiting downstream cellular signaling. Agents that have been studied in this drug class include afatinib and dacomitinib. Both were evaluated and approved in the first-line setting for the treatment of EGFR-mutated NSCLC (Table 2). These agents, however, have not overtaken the first-generation EGFR TKIs as the preferred standard of care. Instead, it is often a physician’s choice as to which agent or drug class best suits a particular patient’s clinical situation [34]. 

Lux-Lung 3 (LL3) compared afatanib to first-line cisplatin and pemetrexed in EGFR-mutated NSCLC. Progression-free survival was improved in the afatanib arm: 11.1 versus 6.9 months, HR 0.58, *p* = 0.001 [15]. Lux-Lung 6 (LL6) later compared afatanib to cisplatin and gemcitabine in an exclusively Asian population. PFS was significantly improved with afatanib: 11.0 months versus 5.6 months, HR 0.28, *p* < 0.0001 [35]. Afatanib and gefitinib were compared 1:1 in a first-line setting in Lux-Lung 7 (LL7). In this setting, the median PFS was similar for both arms: 11.0 versus 10.9 months, HR 0.73, *p* = 0.017. Exploratory analysis suggested an 18 month PFS of 27.3% versus 15.2% and 24 month PFS of 17.6% versus 7.6%, favoring Afatanib [36]. A post hoc analysis of Lux Lung 2 (LL2), LL3, and LL6 assessed the response of afatanib in uncommon EGFR mutations. This analysis confirmed the activity of afatanib in those with exon 18–21 point mutations and duplications. Variants that showed greatest responses included Gly719Xaa (PFS 13.8 months (6.8–not estimable (NE)), overall survival (OS) 26.9 months (16.4–NE)), Leu861Gln( PFS 8.2 months (4.5–16.6), OS 17.1 months (15.3–21.6)), and Ser786Ile (PFS 14.7 months (2.6–NE), OS NE (3.4–NE)) [37]. 

Dacomitinib was compared to gefitinib in a first-line phase III study in patients with EGFR-mutated NSCLC in ARCHER 1050. Dacomitinib was found to improve progression-free survival: 14.7 months versus 9.2 months, HR 0.59, *p* < 0.0001. Higher rates of grade 3–4 events and two treatment-related deaths were reported in the dacomitinib group [38]. A later report of the ARCHER 1050 overall survival (OS) data demonstrated an improvement in OS for dacomitinib: 34.1 versus 26.8 months, HR 0.76, *p* = 0.044 [39]. 

### 3.3. Third-Generation Tyrosine Kinase Inhibitors

Osimertinib is an irreversible inhibitor of T790M resistant and sensitive forms of the EGFR tyrosine kinase. An advantage of osimertinib is its increased selectivity for exon 19, L858R, and T790M mutations over wild-type EGFR, thereby improving the off-target toxicity profile [14]. This agent was evaluated in phase III studies in two different settings: initially, in second-line T790M mutation-positive NSCLC post progression on prior EGFR TKI treatment (AURA3), and subsequently in the first-line setting irrespective of T790M mutation status (FLAURA) [40,41]. AURA3 recruited 419 patients with T790M mutation-positive NSCLC post progression on first-line EGFR TKIs. Patients were randomized in a 2:1 fashion to receive osimertinib or carboplatin and pemetrexed-based chemotherapy. Osmiertinib was found to improve PFS relative to chemotherapy: 10.1 versus 4.4 months, HR 0.30, *p* < 0.001. Central nervous system activity was confirmed with a PFS of 8.5 versus 4.2 months, HR 0.32, favoring osimertinib. Grade 3 and above events were higher in the chemotherapy arm, 23% versus 47% [40]. FLAURA randomly assigned 556 patients to receive either first-line osimertinib or a first-generation EGFR TKI, erlotinib or gefitinib. Median PFS was significantly improved in the experimental arm: 18.9 versus 10.2 months, HR 0.46, *p* < 0.001. Grade 3 and above events were higher in those who received a first-generation TKI, 34% versus 45% [41]. Later analysis of central nervous system (CNS) response rates in the FLAURA study was performed. Two hundred patients in the study had baseline CNS imaging performed at the time of enrolment in the study. Of these, 128 were found to have CNS disease: 61 in the osimertinib arm and 67 in the standard-of-care arm. At the time of reporting, median PFS was not reached in the osimertinib group and was 13.9 months in the standard-of-care group. CNS progression occurred in 20% of the osimertinib arm and 39% of the standard-of-care arm [42]. A phase I study, BLOOM, evaluated the efficacy of osimertinib (160 mg daily) in patients with progressive disease on prior EGFR TKI treatment and confirmed leptomeningeal disease. In an assessment of 23/30 patients with an MRI of the brain at 12 weeks, 10 had radiological improvement and 13 had stable disease. Two of the 23 patients reported a deterioration in their symptoms at the point. Toxicity was manageable with two grade 3 events reported [43].

## 4. EGFR Monotherapy as Adjuvant Therapy in Early-Stage Disease

A number of studies looked at the use of various EGFR inhibitors in the adjuvant setting. Results of these studies were variable and, as such, this strategy has not yet been adopted in routine clinical practice.

The RADIANT study compared the use of adjuvant erlotinib versus placebo in 973 patients who had completely resected stage IB–IIIA stage tumors which expressed EGFR protein by immunohistochemistry or EGFR amplification by fluorescence in situ hybridization. Patients were randomized in a 2:1 fashion to receive erlotinib or placebo over a 2 year period post resection. Adjuvant chemotherapy was allowed if warranted. No difference in disease-free survival (DFS) was observed between the two groups in the intention to treat population: 50.5 months for erlotinib and 48.2 months for placebo, HR 0.90, *p* = 0.324. A subgroup analysis of those with specific EGFR sensitizing mutations revealed a numerical improvement in median DFS: 46.4 months for erlotinib and 28.5 months for placebo; however, this was not statistically significant. At 22% maturity, no OS survival advantage was identified in this cohort, HR 1.09, *p* = 0.815. As observed from this study, the experimental and placebo DFS curves returned together upon discontinuation of EGFR therapy, suggesting no added benefit from the early introduction of erlotinib [44]. Gefitinib was evaluated in the NCIC CTG BR19 study. In total, 503 patients with resected NSCLC, EGFR status unknown, were randomized in a 1:1 fashion to receive gefitinib or placebo for 2 years on completion of started therapy. This study was terminated early due to an unfavorable initial in-term analysis. Median follow-up in this study was 4.7 years. No difference in OS or DFS was observed between arms: OS HR 1.24, *p* = 0.14; DFS HR 1.22, *p* = 0.15. The study concluded that adjuvant gefitinib offered no additional advantage [45]. ADJUVANT/CTONG1104, a phase III study, compared adjuvant gefitinib directly with adjuvant vinorelbine plus cisplatin in patients with resected stage II–IIIA EGFR-mutated NSCLC. In total, 222 patients were randomized 1:1 to received gefitinib for 24 months or vinorelbine plus cisplatin for four cycles. Median DFS was 28.7 months for gefitinib and 18 months for chemotherapy, HR 0.60, *p* = 0.0054. Like the RADIANT study, the DFS curves started to converge at the time of withdrawal of EGFR therapy [46]. Updated OS analysis with a median follow-up of 76.9 months showed no difference between groups: 75.5 months for gefitinib and 79.2 months for chemotherapy, HR 0.96, *p* = 0.823. The 3 and 5 year OS rates were 68.6% and 53.8% for gefitinib and 67.5% and 52.4% for vinerolbine plus cisplatin, respectively [47]. The EVAN phase II study compared adjuvant erlotinib with cisplatin and vinerolbine in 102 patients with resected stage IIIA EGFR-positive NSCLC. This study reported a 2 year DFS in favor of erlotinib: 2 year DFS 81.4%, 95% confidence interval (CI) 69.6–93.1% for erlotinib; 2 year DFS 44.6%, 95% CI 26.9–62.4% for chemotherapy. The authors cautioned that a larger phase III trial was required to verify these results, and this is especially relevant in the context of negative larger phase III studies of first-line EGFR inhibitors [48]. ADAURA evaluated the third-generation EGFR TKI, osimertinib, in patients with resected stage IB–IIIA resected EGFR-mutated NSCLC. Adjuvant chemotherapy was allowed if warranted. In total, 682 patients were randomized to receive osimertinib or placebo on a 1:1 basis for a total of 3 years post resection. In the total population, the 2 year DFS rate was 89% with osimertinib and 53% with placebo, HR 0.2, *p* < 0.0001. OS data at the time of reporting were immature [49]. 

EGFR inhibitors were evaluated in the adjuvant setting post concurrent chemo-radiotherapy in locally advanced stage III disease. SWOG S0023 evaluated gefitinib as a maintenance therapy post completion of cisplatin and etoposide concurrently with radiotherapy and three cycles of docetaxel in an unselected population. This study closed prematurely. OS was inferior in the experimental arm. With a median follow-up of 27 months, OS for gefitinib was 23 months compared to 35 months for placebo, HR 0.633, *p* = 0.013 [50]. The impact of harboring an EGFR mutation was evaluated in a study by Tanaka et al. In this study, the group assessed the effect of EGFR mutation status on progression-free survival in those with stage III NSCLC treated with chemo-radiotherapy. In total, 104 patients were recruited, whose EGFR status was known, while 29 patients (28%) were positive for an EGFR mutation. This study identified shorter progression-free survival in those with EGFR-mutated disease: 9.8 months versus 16.5 months, *p* = 0.041. The shorter PFS was predominantly accounted for due to recurrence at distant sites in the EGFR mutant group [51]. A similar study recruited 197 patients with stage III NSCLC treated with concurrent chemo-radiotherapy (81 EGFR wild type, 36 mutants, and 80 status unknown). This study also reported a shorter PFS in the mutant group compared to those with wild-type disease, predominately due to relapse at distant sites: 8.9 months versus 11.8 months, *p* = 0.013 [52]. 

## 5. Combination Strategies

Combining existing and new treatments with EGFR-targeted therapy is a potential strategy in combating resistance to treatment in non-small-cell lung cancer. Using agents together is attractive due to the potential synergy of mechanisms and the elimination of resistant clones, but this can come at the cost of greater toxicity. Various combinations of EGFR TKI therapy have been investigated including cytotoxic chemotherapy, antiangiogenic agents, and immune checkpoint inhibitors. 

### 5.1. EGFR Inhibitors and Chemotherapy Combinations

The optimal strategy for using EGFR with cytotoxic agents is not known, with rationale provided for intercalated, sequential, and concurrent regimens on the basis of clinical and preclinical data. Initial trials of EGFR-directed therapy saw TKIs paired with chemotherapy. Although the intention to treat population analyses yielded no improvement in efficacy, when the EGFR mutant population was examined subsequently, a superiority emerged [53,54]. Preclinical data suggested synergy between chemotherapeutic agents, for instance, pemetrexed, which, in combination with gefitinib, has actions affecting cell growth and signaling and promotes apoptosis, which was proposed as a potential mechanism to overcome resistance to the TKI [55].

IMPRESS was a phase III trial conducted internationally which randomized 265 patients to receive either gefitinib with up to six cycles of cisplatin plus pemetrexed or placebo with the same chemotherapy regime following progression of disease on first-line gefitinib. Oral therapy continued following the completion of chemotherapy. Patients were tested for but not stratified by presence of T790M mutation. After a median of 11.2 months of follow-up, the median number of chemotherapy cycles received in both groups was five. Progression was seen in 74% of the patients in the gefitinib compared to 81% in the placebo group with a median PFS of 5.4 months in both groups, HR 0.86, *p* < 0.27 [56]. Median overall survival was reported in favor of the placebo at 14.8 vs. 17.2 months, HR 1.62, *p* < 0.03, and updated in a follow-up publication at 13.4 vs. 19.5 months, HR 1.44, *p* = 0.016, favoring placebo [9]. Adverse events were common, but generally grade 1–2 in both groups. Diarrhea, stomatitis, vomiting, and neutropenia were seen more frequently in the group receiving gefitinib. 

In the first-line setting, there were three studies that published data using combinations of cytotoxic chemotherapy and EGFR-directed treatment. Han et al. conducted a head-to-head study of four cycles of carboplatin/pemetrexed doublet chemotherapy combined with gefitinib on days 5–21 of a 3 week cycle, followed by maintenance gefitinib with standard chemotherapy and gefitinib alone. In total, 121 patients were randomized 1:1:1 with a primary outcome of PFS. Efficacy results were favorable toward the combination, with mPFS reported as 17.5 (15.3–19.7), 11.9 (9.1–14.6), and 5.7 (5.2–6.3) in the triplet, gefitinib alone, and doublet groups, respectively. The hazard ratio for the triplet vs. gefitinib alone was 0.48, *p* < 0.003, and that vs. chemotherapy was 0.16, *p* < 0.001 [57]. Toxicities experienced by patients were higher with the triplet, whereby 10% of patients experienced grade 3–4 liver dysfunction. Similar numbers also experienced rash in those patients who received gefitinib, either alone or in combination. 

Noronha and colleagues reported similar results from a phase III trial which enrolled 350 patients to receive gefitinib alone or with carboplatin/pemetrexed chemotherapy. PFS from this trial at a follow-up of 17 months was 16 (13.5–18.5) months vs. 8 (7.0–9.0) months, HR 0.5, *p* < 0.001 with overall survival not yet reached [58]. This improvement in PFS was accompanied by significantly more toxicity including an 11% rate of nephrotoxicity, G3/4 anemia in 19% vs. 1%, neutropenia 16% vs. 0%, and diarrhea 14% vs. 9%. In addition, G3/4 hyponatremia was seen in 24% and 16% of patients and hypertension was seen in 26% and 24% of patients [58]. 

Most recently, results from the NEJ009 study were published, which looked at 345 patients again treated with gefitinib alone or with carboplatin/pemetrexed. This was a multicenter trial, in a Japanese population, which followed a phase II trial suggesting that concurrent chemotherapy with gefitinib was superior to sequential alternating treatment in NEJ009; PFS was again in favor of the combination group at 20.9 (17.9–24.2) months vs. 11.1 (8.97–13.4) months, HR 0.49, *p* < 0.001 [59,60]. This trial also showed a benefit in terms of overall survival; however, again, toxicities were greater. Grade 3 or higher myelotoxicity in 20–30% of patients was seen in the combination group, while liver dysfunction was also seen; interestingly, this only occurred in 22% of those receiving gefitinib monotherapy and in 12% of those receiving the combination (Table 3).

An alternative strategy is with an intercalated regime as described by Wu and colleagues reporting the results of FASTACT-2. Patients were randomized to receive six cycles of gemcitabine with platinum every 4 weeks with either erlotinib or placebo administered on Days 15–28 of the cycle. In patients with activating EGFR mutations, results were statistically favored toward the combination therapy showing PFS 16.8 months (12.9–20.4) vs. 6.9 months (5.3–7.6), HR 0.25, *p* < 0.001 and median OS 31.4 months (22.2–NR) vs. 20.6 months (14.2–26.9), HR 0.48, *p* < 0.0092. Of note, the toxicity appeared to be similar, with rates of neutropenia grade 3 or higher being 29% vs. 25%, thrombocytopenia 14% in both groups, and anemia 12% vs. 9% [61]. Finally, a further study currently recruiting and yet to report, FLAURA2, is investigating the second-generation EGFR TKI osimertinib as monotherapy or in combination [62].

### 5.2. EGFR Inhibitor and Antiangiogenic Combinations

Vascular endothelial growth factor (VEGF) in lung cancer is considered an attractive target for therapy due to the role this pathway plays in lung cancer growth and progression [63]. VEGF monoclonal antibodies such as bevacizumab are used in combination with chemotherapy as routine practice in many countries. Combining anti-EGFR therapy and VEGF-directed antibodies may have acceptable toxicity, as these agents historically have differing toxicity profiles and, therefore, overlap should be rare. In addition, it was shown that the combination of anti-EGFR therapy and antiangiogenesis agents delays or suppresses the activation of T790M resistance mutations [64,65].

NEJ026 compared the combination of bevacizumab 15 mg/kg every 3 weeks plus erlotinib 150 mg daily with erlotinib 150 mg monotherapy in a Japanese population, with a primary end point of progression-free survival. At the publication of the first interim analysis, 228 patients were randomized with a median follow-up of 12.4 months. Median PFS was 16.9 months (14.2–21) vs. 13.3 months (11.1–15.3), HR 0.61, *p* = 0.016 in favor of the combined and monotherapy arm. Objective response was 81% vs. 74%. Grade 3 or above adverse events in the intervention group were 88% vs. 46% in the group receiving erlotinib with rash seen in 21% of patients in either cohort. Hypertension, proteinuria, and nonpulmonary hemorrhage occurred at higher rates for the combination [66]. The “bevacizumab plus erlotinib study” (BEVERLY) also investigated this combination with no results published to date [67], whilst SPIRAL II is looking at the combination of bevacizumab and osimertinib [68].

In the phase III RELAY study, 449 mainly Asian (77%) patients, with EGFR mutations and no CNS metastases, were randomized to receive erlotinib 150 mg + ramucirumab 10 mg/kg every 2 weeks versus erlotinib 150 mg daily and placebo. Initial results, after a median follow-up period of 20.7 months, supported the combination with a median PFS of 19.4 months vs. 12.4 months, HR 0.591, *p* < 0.0001, and an objective response rate of 76.3% vs. 74.7%. The median OS was not yet reached in either arm. Grade 3 toxicities were reported to be 72% for the combined arm against 54% in the control arm and consisted mainly of hypertension (24% vs. 5%). There was one treatment-related death from hemothorax in the intervention arm [69].

### 5.3. EGFR Inhibitors and Immunotherapy Combinations

Combining EGFR and immune checkpoint therapies was explored in a limited number of trials. The only phase 3 trial was CAURAL, which combined osimertinib with durvalumab in a population who had confirmed progression on EGFR TKI therapy, with T790M mutation-positive disease. This trial was ceased early due to safety concerns from an earlier phase Ib trial looking at the same combination which showed rates of interstitial lung disease to be 38%. At the termination of the study, 29 patients were recruited. Reported adverse events included diarrhea in 53% rash in 67% of patients, and, at the time of reporting, no patients in the combination group had developed interstitial lung disease. Response rates were higher in the monotherapy arm at 80% compared to 64% with the combination, although these results cannot be considered as formal analysis. No further phase III trials have reported results to our knowledge to date [70].

## 6. Evolving Role of Liquid Biopsy in NSCLC

Noninvasive means of assessing tumoral information in lung cancers is becoming an increasingly important factor given that tumor tissue samples are often limiting [71]. The ability to assess this from blood samples is desirable as it is (1) noninvasive, (2) can be repeated to assess dynamically changing tumoral status, and (3) can be used to monitor a patient over the course of disease by serial blood sampling to determine treatment efficacy in a real-time manner. The ability to detect minimal residual disease (MRD) after curative surgery, as well as the potential to identify patients that are likely to develop recurrences, remains an unmet clinical need.

A number of studies highlighted the use of circulating tumor DNA (ctDNA), circulating tumor cells (CTCs), and exosomes which may be used for a liquid biopsy [72,73]. Circulating tumor cells are thought to be the “metastatic seeds” in circulation and represent single, intact cancer cells or cell clusters which travel transiently in the blood to seed at distant sites. Molecular characterization of CTCs has revealed that genomic changes of the tumor can be readily assessed via CTCs. This was recently highlighted by a number of studies identifying translocations/mutations in anaplastic lymphoma kinase (ALK) and EGFR in CTCs [74,75,76,77,78]. For EGFR mutations, the sensitivity of the assay increased with higher numbers of detected CTCs with concordance between EGFR mutation status in the tumor and CTC samples [77].

Whilst enumeration of CTCs was found to be prognostic across a number of tumor types including lung cancers, CTC clusters appear to play a more important in forming overt metastasis. CTC clusters, which are composed of cancer cells and neutrophils, appear to have immune-evasion mechanisms to protect them from being destroyed in the blood [79,80]. Moreover, recent studies highlighted how CTC clusters have a 23–50-fold higher metastatic capacity compared to single CTCs, highlighting the importance of understanding these cell clusters, as well as identifying strategies to disrupt CTC cluster-mediated metastasis [81,82]. 

Circulating tumor DNA (ctDNA) is thought to mainly be released by apoptotic cells and carry tumor-specific DNA, which, in principle, may match that of the primary tumor. Recent studies highlighted that ctDNA is readily detectable in the plasma with concordant mutations to that of the primary tissue [83]. In evaluating EGFR T790M mutations across 21 studies and 1639 patients, the pooled sensitivity of ctDNA analysis was 0.67 (0.64–0.70) and the pooled specificity was 0.85 (0.82–0.87). The pooled negative positive predictive value (PPV) was 0.85 (0.82–0.87) and the pooled negative predictive value (NPV) was 0.60 (0.56–0.63). The reported area under the curve (AUC) of the summary receiver operating curve (sROC) was 0.77. This data highlighted the promising nature of this noninvasive approach and the need for standardized methodologies and clinical validation [84].

Moreover, studies have demonstrated that ctDNA can be used to detect minimal residual disease (MRD) after surgical resection and, therefore, identify patients at an increased risk of disease recurrence ahead of standard-of-care imaging surveillance [85]. With the recent data from the TRACERx (tracking non-small-cell lung cancer evolution through therapy (Rx)) study available, independent predictors of ctDNA release and phylogenetic ctDNA tracking provided insights into the subclonal nature of lung cancer relapse and metastasis [86].

## 7. Conclusions

Patients with advanced or inoperable EGFR-mutated NSCLC have an array of options at their disposal to treat their disease. However, even with the significant advances achieved to date, resistance has evolved that ultimately overcomes a drug’s inhibitory effects. EGFR TKIs used as monotherapy remains the recommended standard of care. Ongoing efforts are underway to establish the best sequence for these agents and determine if combination strategies with other anticancer treatments may offer better disease control. Liquid biopsy with analysis of circulating tumor cells may offer a minimally invasive approach that could be readily repeated at multiple time points over the course of treatment. Such a strategy may offer timely data prior to overt radiologic progression and/or yield early insight into evolving resistance mechanisms to a specific therapeutic agent in use at that time.

## Figures and Tables

**Table 1:** Outcome of first-generation epidermal growth factor receptor (EGFR) tyrosine kinase inhibitors in the first-line setting. PFS, progression-free survival.

Study	EGFR Inhibitor PFS (Months)	Chemotherapy PFS (Months)
Erlotinib versus carboplatin/gemcitabine (OPTIMAL)	13.1	4.6
Erlotinib versus platinum doublet (EURTAC)	9.7	5.2
Erlotinib versus cisplatin/gemcitabine (ENSURE)	11	5.5
Gefitinib versus cisplatin/docetaxel (WJTOG3405)	9.2	6.3
Gefitinib versus carboplatin/paclitaxel	10.8	5.4
Gefitinib versus carboplatin/paclitaxel (IPASS)	9.5	6.3

**Table 2:** Outcomes of second-generation EGFR tyrosine kinase inhibitors in the first-line setting. LL, Lux-Lung.

Study	EGFR Inhibitor PFS (Months)	Chemotherapy PFS (Months)
Afatanib versus cisplatin/pemetrexed (LL3)	11.1	6.9
Afatanib versus cisplatin/gemcitabine (LL6)	11	5.6
Afatanib versus gefitinib (LL7)	11	10.9
Dacomitinib versus gefitinib (ARCHER 1050)	14.7	9.2

**Table 3:** Results of phase III trials comparing EGFR TKIs in combination with EGFR TKI alone. OS, overall survival.

Study	Intervention	No	mPFS (Months)	mOS (Months)	ORR (%)
	Chemotherapy and anti-EGFR				
IMPRESS	gefitinib, cisplatin, pemetrexed vs. placebo, cisplatin, pemetrexed	265	5.4 vs. 5.4 HR 0.86 *p* < 0.27	14.8 vs. 17.2 HR 1.62 *p* < 0.03	32 vs. 34
Han et al.	gefitinib, carboplatin, pemetrexed vs. gefitinib vs. carboplatin, pemetrexed	121	17.5 vs. 11.9 vs. 5.7 HR 0.16 *P* < 0.001	32.6 vs. 25.8 vs. 24.3 HR 0.46 *p* < 0.016	82.5 vs. 65.9 vs. 32.5
NEJ009	gefitinib, carboplatin, pemetrexed and maintenance pemetrexed vs. gefitinib	344	20.9 vs. 11.2 HR 0.49 *p* < 0.001	52.2 vs. 38.8 HR 0.70 *p* < 0.013	
Noronha et al.	gefitinib, carboplatin, pemetrexed and maintenance pemetrexed vs. gefitinib	350	16 vs. 8 HR 0.5 *p* < 0.001	NR vs. 18 HR 0.45 *p* < 0.001	81 vs. 69
FASTACT-2 (EGFR subpopulation)	erlotinib, platinum, gemcitabine vs. erlotinib		16.8 vs. 6.9, HR 0.25 *p* < 0.0001	31.4 vs. 20.6, HR 0.48 *p* < 0.0092	76.3 vs. 74.7
	Antiangiogenesis agents and anti-EGFR				
NEJ026	erlotinib, bevacizumab vs. erlotinib	228	16.9 vs. 13.3 HR 0.61 *p* < 0.016	NR vs. NR	81% vs. 74%
RELAY	erlotinib, ramucirumab vs. erlotinib, placebo	449	19.4 vs. 12.4 months HR 0.59 *p* < 0.0001	NR vs. NR	76.3 vs. 74.7
	Immunotherapy and anti-EGFR				
CAURAL	osimertinib vs. osimertinib, durvalumab	29	Nil	Nil	Nil

## References

[1] Gower A., Wang Y., Giaccone G. (2014). Oncogenic drivers, targeted therapies, and acquired resistance in non-small-cell lung cancer. J. Mol. Med..

[2] Hensing T., Chawla A., Batra R., Salgia R. (2013). A Personalized Treatment for Lung Cancer: Molecular Pathways, Targeted Therapies, and Genomic Characterization. Adv. Exp. Med. Biol..

[3] Jiang W., Cai G., Hu P.C., Wang Y. (2018). Personalized medicine in non-small cell lung cancer: A review from a pharmacogenomics perspective. Acta Pharm. Sin. B.

[4] Rosell R., Moran T., Queralt C., Porta R., Cardenal F., Camps C., Majem M., López-Vivanco G., Isla D., Provencio M. (2009). Screening for Epidermal Growth Factor Receptor Mutations in Lung Cancer. N. Engl. J. Med..

[5] Yun C.-H., Boggon T.J., Li Y., Woo M.S., Greulich H., Meyerson M., Eck M.J. (2007). Structures of Lung Cancer-Derived EGFR Mutants and Inhibitor Complexes: Mechanism of Activation and Insights into Differential Inhibitor Sensitivity. Cancer Cell.

[6] Shigematsu H., Lin L., Takahashi T., Nomura M., Suzuki M., Wistuba I.I., Fong K.M., Lee H., Toyooka S., Shimizu N. (2005). Clinical and Biological Features Associated with Epidermal Growth Factor Receptor Gene Mutations in Lung Cancers. J. Natl. Cancer Inst..

[7] Barker A.J., Gibson K., Grundy W., Godfrey A.A., Barlow J.J., Healy M.P., Woodburn J.R., Ashton E.S., Curry B.J., Scarlett L. (2001). Studies leading to the identification of ZD1839 (IRESSA): An orally active, selective epidermal growth factor receptor tyrosine kinase inhibitor targeted to the treatment of cancer. Bioorganic Med. Chem. Lett..

[8] Moyer J.D., Barbacci E.G., Iwata K.K., Arnold L., Boman B., Cunningham A., Diorio C., Doty J., Morin M.J., Moyer M.P. (1997). Induction of apoptosis and cell cycle arrest by CP-358,774, an inhibitor of epidermal growth factor receptor tyrosine kinase. Cancer Res..

[9] Mok T.S.-K., Kim S.-W., Wu Y.-L., Nakagawa K., Yang J.-J., Ahn M.-J., Wang J., Yang J.C., Lu Y., Atagi S. (2017). Gefitinib Plus Chemotherapy Versus Chemotherapy in Epidermal Growth Factor Receptor Mutation–Positive Non–Small-Cell Lung Cancer Resistant to First-Line Gefitinib (IMPRESS): Overall Survival and Biomarker Analyses. J. Clin. Oncol..

[10] Pao W., Miller A.V., Politi A.K., Riely G.J., Somwar R., Zakowski M.F., Kris M.G., Varmus H. (2005). Acquired Resistance of Lung Adenocarcinomas to Gefitinib or Erlotinib Is Associated with a Second Mutation in the EGFR Kinase Domain. PLoS Med..

[11] Yu H.A., Tian S.K., Drilon A.E., Borsu L., Riely G.J., Arcila M.E., Ladanyi M. (2015). Acquired Resistance ofEGFR-Mutant Lung Cancer to a T790M-Specific EGFR Inhibitor. JAMA Oncol..

[12] Shimamura T., Ji H., Minami Y., Thomas R.K., Lowell A.M., Shah K., Greulich H., Glatt K.A., Meyerson M., Shapiro G.I. (2006). Non–Small-Cell Lung Cancer and Ba/F3 Transformed Cells Harboring the ERBB2 G776insV_G/C Mutation Are Sensitive to the Dual-Specific Epidermal Growth Factor Receptor and ERBB2 Inhibitor HKI-272. Cancer Res..

[13] Wang S.E., Narasanna A., Perez-Torres M., Xiang B., Wu F.Y., Yang S., Carpenter G., Gazdar A.F., Muthuswamy S.K., Arteaga C.L. (2006). HER2 kinase domain mutation results in constitutive phosphorylation and activation of HER2 and EGFR and resistance to EGFR tyrosine kinase inhibitors. Cancer Cell.

[14] Cross D.A.E., Ashton S.E., Ghiorghiu S., Eberlein C., Nebhan C.A., Spitzler P.J., Orme J.P., Finlay M.R.V., Ward R.A., Mellor M.J. (2014). AZD9291, an Irreversible EGFR TKI, Overcomes T790M-Mediated Resistance to EGFR Inhibitors in Lung Cancer. Cancer Discov..

[15] Sequist L.V., Yang J.C., Yamamoto N., O’Byrne K., Hirsh V., Mok T.S.-K., Geater S.L., Orlov S.V., Tsai C.-M., Boyer M. (2013). Phase III Study of Afatinib or Cisplatin Plus Pemetrexed in Patients with Metastatic Lung Adenocarcinoma With EGFR Mutations. J. Clin. Oncol..

[16] Solca F., Dahl G., Zoephel A., Bader G., Sanderson M., Klein C., Krämer O.H., Himmelsbach F., Haaksma E., Adolf G.R. (2012). Target Binding Properties and Cellular Activity of Afatinib (BIBW 2992), an Irreversible ErbB Family Blocker. J. Pharm. Exp..

[17] Miller A.V., Hirsh V., Cadranel J., Chen Y.-M., Park K., Kim S.W., Zhou C., Su W.-C., Wang M., Sun Y. (2012). Afatinib versus placebo for patients with advanced, metastatic non-small-cell lung cancer after failure of erlotinib, gefitinib, or both, and one or two lines of chemotherapy (LUX-Lung 1): A phase 2b/3 randomised trial. Lancet Oncol..

[18] Murakami H., Tamura T., Takahashi T., Nokihara H., Naito T., Nakamura Y., Nishio K., Seki Y., Sarashina A., Shahidi M. (2011). Phase I study of continuous afatinib (BIBW 2992) in patients with advanced non-small cell lung cancer after prior chemotherapy/erlotinib/gefitinib (LUX-Lung 4). Cancer Chemother. Pharm..

[19] Yang J.C., Shih J.-Y., Su W.-C., Hsia T.-C., Tsai C.-M., Ou S.-H.I., Yu C.-J., Chang G.-C., Ho C.-L., Sequist L.V. (2012). Afatinib for patients with lung adenocarcinoma and epidermal growth factor receptor mutations (LUX-Lung 2): A phase 2 trial. Lancet Oncol..

[20] Graham R.P., Treece A.L., Lindeman N.I., Vasalos P., Jennings L.J., Rimm D.L., Shan M. (2018). Worldwide Frequency of Commonly Detected EGFR Mutations. Arch. Pathol. Lab. Med..

[21] Balak M.N., Gong Y., Riely G.J., Somwar R., Li A.R., Zakowski M.F., Chiang A., Yang G., Ouerfelli O., Kris M.G. (2006). Novel D761Y and Common Secondary T790M Mutations in Epidermal Growth Factor Receptor-Mutant Lung Adenocarcinomas with Acquired Resistance to Kinase Inhibitors. Clin. Cancer Res..

[22] Kosaka T., Yatabe Y., Endoh H., Yoshida K., Hida T., Tsuboi M., Tada H., Kuwano H., Mitsudomi T. (2006). Analysis of Epidermal Growth Factor Receptor Gene Mutation in Patients with Non-Small Cell Lung Cancer and Acquired Resistance to Gefitinib. Clin. Cancer Res..

[23] Yun C.-H., Mengwasser K.E., Toms A.V., Woo M.S., Greulich H., Wong K.-K., Meyerson M., Eck M.J. (2008). The T790M mutation in EGFR kinase causes drug resistance by increasing the affinity for ATP. Proc. Natl. Acad. Sci. USA.

[24] Chabon J.J., Simmons A.D., Lovejoy A.F., Esfahani M.S., Newman A.M., Haringsma H.J., Kurtz D.M., Stehr H., Scherer F., Karlovich C.A. (2016). Circulating tumour DNA profiling reveals heterogeneity of EGFR inhibitor resistance mechanisms in lung cancer patients. Nat. Commun..

[25] Thress K.S., Paweletz C.P., Felip E., Cho B.C., Stetson D., Dougherty B., Lai Z., Markovets A., Vivancos A., Kuang Y. (2015). Acquired EGFR C797S mutation mediates resistance to AZD9291 in non–small cell lung cancer harboring EGFR T790M. Nat. Med..

[26] Bersanelli M., Minari R., Bordi P., Gnetti L., Bozzetti C., Squadrilli A., Lagrasta C.A.M., Bottarelli L., Osipova G., Capelletto E. (2016). L718Q Mutation as New Mechanism of Acquired Resistance to AZD9291 in EGFR -Mutated NSCLC. J. Thorac. Oncol..

[27] Ercan D., Choi H.G., Yun C.-H., Capelletti M., Xie T., Eck M.J., Gray N.S., Jänne P.A. (2015). EGFR Mutations and Resistance to Irreversible Pyrimidine-Based EGFR Inhibitors. Clin. Cancer Res..

[28] Zheng D., Hu M., Bai Y., Zhu X., Lu X., Wu C., Wang J., Liu L., Wang Z., Ni J. (2017). EGFR G796D mutation mediates resistance to osimertinib. Oncotarget.

[29] Zhou C., Wu Y.-L., Chen G., Feng J., Liu X.-Q., Wang C., Zhang S., Wang J., Zhou S., Ren S. (2011). Erlotinib versus chemotherapy as first-line treatment for patients with advanced EGFR mutation-positive non-small-cell lung cancer (OPTIMAL, CTONG-0802): A multicentre, open-label, randomised, phase 3 study. Lancet Oncol..

[30] Rosell R., Carcereny E., Gervais R., Vergnenègre A., Massuti B., Felip E., Palmero R., Garcia-Gomez R., Pallares C., Sanchez J.M. (2012). Erlotinib versus standard chemotherapy as first-line treatment for European patients with advanced EGFR mutation-positive non-small-cell lung cancer (EURTAC): A multicentre, open-label, randomised phase 3 trial. Lancet Oncol..

[31] Wu Y.-L., Zhou C., Liam C.-K., Wu G., Liu X., Zhong Z., Lu S., Cheng Y., Han B., Chen L. (2015). First-line erlotinib versus gemcitabine/cisplatin in patients with advanced EGFR mutation-positive non-small-cell lung cancer: Analyses from the phase III, randomized, open-label, ENSURE study. Ann. Oncol..

[32] Mitsudomi T., Morita S., Yatabe Y., Negoro S., Okamoto I., Tsurutani J., Seto T., Satouchi M., Tada H., Hirashima T. (2010). Gefitinib versus cisplatin plus docetaxel in patients with non-small-cell lung cancer harbouring mutations of the epidermal growth factor receptor (WJTOG3405): An open label, randomised phase 3 trial. Lancet Oncol..

[33] Wu Y.-L., Saijo N., Thongprasert S., Yang J.C., Han B., Margono B., Chewaskulyong B., Sunpaweravong P., Ohe Y., Ichinose Y. (2017). Efficacy according to blind independent central review: Post-hoc analyses from the phase III, randomized, multicenter, IPASS study of first-line gefitinib versus carboplatin/paclitaxel in Asian patients with EGFR mutation-positive advanced NSCLC. Lung Cancer.

[34] NCCN.org (2018). NCCN Clinical Practice Guidelines in Oncology (NCCN Guidelines®) Non-Small Cell Lung Cancer. NCCN Evidence Blocks. J. Nat. Compr. Cancer Netw..

[35] Wu Y.-L., Zhou C., Hu C.-P., Feng J., Lu S., Huang Y., Li W., Hou M., Shi J.H., Lee K.Y. (2014). Afatinib versus cisplatin plus gemcitabine for first-line treatment of Asian patients with advanced non-small-cell lung cancer harbouring EGFR mutations (LUX-Lung 6): An open-label, randomised phase 3 trial. Lancet Oncol..

[36] Park K., Tan E.-H., O’Byrne K., Zhang L., Boyer M., Mok T., Hirsh V., Yang J.C.-H., Lee K.H., Lu S. (2016). Afatinib versus gefitinib as first-line treatment of patients with EGFR mutation-positive non-small-cell lung cancer (LUX-Lung 7): A phase 2B, open-label, randomised controlled trial. Lancet Oncol..

[37] Yang J.C., Sequist L.V., Geater S.L., Tsai C.-M., Mok T.S.-K., Schuler M., Yamamoto N., Yu C.-J., Ou I.S.-H., Zhou C. (2015). Clinical activity of afatinib in patients with advanced non-small-cell lung cancer harbouring uncommon EGFR mutations: A combined post-hoc analysis of LUX-Lung 2, LUX-Lung 3, and LUX-Lung 6. Lancet Oncol..

[38] Wu Y.-L., Cheng Y., Zhou X., Lee K.H., Nakagawa K., Niho S., Tsuji F., Linke R., Rosell R., Corral J. (2017). Dacomitinib versus gefitinib as first-line treatment for patients with EGFR-mutation-positive non-small-cell lung cancer (ARCHER 1050): A randomised, open-label, phase 3 trial. Lancet Oncol..

[39] Mok T.S.-K., Cheng Y., Zhou X., Lee K.H., Nakagawa K., Niho S., Lee M., Linke R., Rosell R., Corral J. (2018). Improvement in Overall Survival in a Randomized Study That Compared Dacomitinib With Gefitinib in Patients With Advanced Non–Small-Cell Lung Cancer and EGFR-Activating Mutations. J. Clin. Oncol..

[40] Mok T.S., Wu Y.-L., Ahn M.-J., Garassino M.C., Kim H.R., Ramalingam S.S., Shepherd F.A., He Y., Akamatsu H., Theelen W.S. (2016). Osimertinib or Platinum-Pemetrexed in EGFR T790M-Positive Lung Cancer. N. Engl. J. Med..

[41] Soria J.-C., Ohe Y., Vansteenkiste J., Reungwetwattana T., Chewaskulyong B., Lee K.H., Dechaphunkul A., Imamura F., Nogami N., Kurata T. (2018). Osimertinib in UntreatedEGFR-Mutated Advanced Non–Small-Cell Lung Cancer. N. Engl. J. Med..

[42] Reungwetwattana T., Nakagawa K., Cho B.C., Cobo M., Cho E.K., Bertolini A., Bohnet S., Zhou C., Lee K.H., Nogami N. (2018). CNS Response to Osimertinib Versus Standard Epidermal Growth Factor Receptor Tyrosine Kinase Inhibitors in Patients With Untreated EGFR-Mutated Advanced Non–Small-Cell Lung Cancer. J. Clin. Oncol..

[43] Yang J.C., Cho B.C., Kim D.-W., Kim S.W., Lee J., Su W.-C., John T., Kao S.C.-H., Natale R., Goldman J.W. (2017). Osimertinib for patients (pts) with leptomeningeal metastases (LM) from EGFR-mutant non-small cell lung cancer (NSCLC): Updated results from the BLOOM study. J. Clin. Oncol..

[44] Kelly K., Altorki N.K., Eberhardt W.E., O’Brien M.E., Spigel D.R., Crinó L., Tsai C.-M., Kim J.-H., Cho E.K., Hoffman P.C. (2015). Adjuvant Erlotinib Versus Placebo in Patients With Stage IB-IIIA Non–Small-Cell Lung Cancer (RADIANT): A Randomized, Double-Blind, Phase III Trial. J. Clin. Oncol..

[45] Goss G., O’Callaghan C., Lorimer I., Tsao M.-S., Masters G.A., Jett J., Edelman M.J., Lilenbaum R., Choy H., Khuri F. (2013). Gefitinib Versus Placebo in Completely Resected Non–Small-Cell Lung Cancer: Results of the NCIC CTG BR19 Study. J. Clin. Oncol..

[46] Zhong W.-Z., Xu S., Yan H.-H., Yang X.-N., Zhou Q., Wu Y.-L., Wang Q., Mao W.-M., Wu L., Shen Y. (2018). Gefitinib versus vinorelbine plus cisplatin as adjuvant treatment for stage II–IIIA (N1–N2) EGFR-mutant NSCLC (ADJUVANT/CTONG1104): A randomised, open-label, phase 3 study. Lancet Oncol..

[47] Wu Y.-L., Zhong W., Wang Q., Mao W., Xu S.-T., Wu L., Chen C., Cheng Y., Xu L., Wang J. (2020). CTONG1104: Adjuvant gefitinib versus chemotherapy for resected N1-N2 NSCLC with EGFR mutation—Final overall survival analysis of the randomized phase III trial 1 analysis of the randomized phase III trial. J. Clin. Oncol..

[48] Yue D., Xu S., Wang Q., Li X., Shen Y., Zhao H., Chen C., Mao W., Liu W., Liu J. (2018). Erlotinib versus vinorelbine plus cisplatin as adjuvant therapy in Chinese patients with stage IIIA EGFR mutation-positive non-small-cell lung cancer (EVAN): A randomised, open-label, phase 2 trial. Lancet Respir. Med..

[49] Herbst R.S., Tsuboi M., John T., Grohé C., Majem M., Goldman J.W., Kim S.-W., Marmol D., Rukazenkov Y., Wu Y.-L. (2020). Osimertinib as adjuvant therapy in patients (pts) with stage IB–IIIA EGFR mutation positive (EGFRm) NSCLC after complete tumor resection: ADAURA. J. Clin. Oncol..

[50] Kelly K., Chansky K., Gaspar L.E., Albain K.S., Jett J., Ung Y.C., Lau D.H., Crowley J.J., Gandara D.R. (2008). Phase III Trial of Maintenance Gefitinib or Placebo After Concurrent Chemoradiotherapy and Docetaxel Consolidation in Inoperable Stage III Non–Small-Cell Lung Cancer: SWOG S0023. J. Clin. Oncol..

[51] Tanaka K., Hida T., Oya Y., Oguri T., Yoshida T., Shimizu J., Horio Y., Hata A., Kaji R., Fujita S. (2015). EGFR Mutation Impact on Definitive Concurrent Chemoradiation Therapy for Inoperable Stage III Adenocarcinoma. J. Thorac. Oncol..

[52] Park S.E., Noh J.M., Kim Y.J., Lee H.S., Cho J.H., Lim S.W., Ahn Y.C., Pyo H., Choi Y.-L., Han J. (2018). EGFR Mutation Is Associated with Short Progression-Free Survival in Patients with Stage III Non-squamous Cell Lung Cancer Treated with Concurrent Chemoradiotherapy. Cancer Res. Treat..

[53] Herbst R.S., Prager D., Hermann R., Fehrenbacher L., Johnson B.E., Sandler A.B., Kris M.G., Tran H.T., Klein P., Li X. (2005). TRIBUTE: A Phase III Trial of Erlotinib Hydrochloride (OSI-774) Combined With Carboplatin and Paclitaxel Chemotherapy in Advanced Non–Small-Cell Lung Cancer. J. Clin. Oncol..

[54] Gatzemeier U., Pluzanska A., Szczesna A., Kaukel E., Roubec J., De Rosa F., Milanowski J., Karnicka-Mlodkowski H., Pešek M., Serwatowski P. (2007). Phase III Study of Erlotinib in Combination With Cisplatin and Gemcitabine in Advanced Non–Small-Cell Lung Cancer: The Tarceva Lung Cancer Investigation Trial. J. Clin. Oncol..

[55] Wu M., Yuan Y., Pan Y.-Y., Zhang Y. (2014). Combined gefitinib and pemetrexed overcome the acquired resistance to epidermal growth factor receptor tyrosine kinase inhibitors in non-small cell lung cancer. Mol. Med. Rep..

[56] Soria J.-C., Wu Y.-L., Nakagawa K., Kim S.W., Yang J.-J., Ahn M.-J., Wang J., Yang J.C., Lu Y., Atagi S. (2015). Gefitinib plus chemotherapy versus placebo plus chemotherapy in EGFR-mutation-positive non-small-cell lung cancer after progression on first-line gefitinib (IMPRESS): A phase 3 randomised trial. Lancet Oncol..

[57] Han B., Jin B., Chu T., Niu Y., Dong Y., Xu J., Gu A., Zhong H., Wang H., Zhang X. (2017). Combination of chemotherapy and gefitinib as first-line treatment for patients with advanced lung adenocarcinoma and sensitive EGFR mutations: A randomized controlled trial. Int. J. Cancer.

[58] Noronha V., Patil V.M., Joshi A., Menon N., Chougule A., Mahajan A., Janu A., Purandare N., Kumar R., More S. (2020). Gefitinib Versus Gefitinib Plus Pemetrexed and Carboplatin Chemotherapy in EGFR-Mutated Lung Cancer. J. Clin. Oncol..

[59] Oizumi S., Sugawara S., Minato K., Harada T., Inoue A., Fujita Y., Maemondo M., Watanabe S., Ito K., Gemma A. (2018). Updated survival outcomes of NEJ005/TCOG0902: A randomised phase II study of concurrent versus sequential alternating gefitinib and chemotherapy in previously untreated non-small cell lung cancer with sensitive EGFR mutations. Esmo Open.

[60] Hosomi Y., Morita S., Sugawara S., Kato T., Fukuhara T., Gemma A., Takahashi K., Fujita Y., Harada T., Minato K. (2019). Gefitinib Alone Versus Gefitinib Plus Chemotherapy for Non-Small-Cell Lung Cancer with Mutated Epidermal Growth Factor Receptor: NEJ009 Study. J. Clin. Oncol..

[61] Wu Y.-L., Lee J.S., Thongprasert S., Yu C.-J., Zhang L., Ladrera G., Srimuninnimit V., Sriuranpong V., Sandoval-Tan J., Zhu Y. (2013). Intercalated combination of chemotherapy and erlotinib for patients with advanced stage non-small-cell lung cancer (FASTACT-2): A randomised, double-blind trial. Lancet Oncol..

[62] Jänne P., Planchard D., Howarth P., Todd A., Kobayashi K. (2019). OA07.01 Osimertinib Plus Platinum/Pemetrexed in Newly-Diagnosed Advanced EGFRm-Positive NSCLC.; The Phase 3 FLAURA2 Study. J. Thorac. Oncol..

[63] Hall R.D., Le T.M., Haggstrom D.E., Gentzler R.D. (2015). Angiogenesis inhibition as a therapeutic strategy in non-small cell lung cancer (NSCLC). Transl. Lung Cancer Res..

[64] Ichihara E., Ohashi K., Takigawa N., Osawa M., Ogino A., Tanimoto M., Kiura K. (2009). Effects of Vandetanib on Lung Adenocarcinoma Cells Harboring Epidermal Growth Factor Receptor T790M Mutation In vivo. Cancer Res..

[65] Naumov G.N., Nilsson M.B., Cascone T., Briggs A., Straume O., Akslen L.A., Lifshits E., Byers L.A., Xu L., Wu H.-K. (2009). Combined vascular endothelial growth factor receptor and epidermal growth factor receptor (EGFR) blockade inhibits tumor growth in xenograft models of EGFR inhibitor resistance. Clin. Cancer Res..

[66] Saito H., Fukuhara T., Furuya N., Watanabe K., Sugawara S., Iwasawa S., Tsunezuka Y., Yamaguchi O., Okada M., Yoshimori K. (2019). Erlotinib plus bevacizumab versus erlotinib alone in patients with EGFR-positive advanced non-squamous non-small-cell lung cancer (NEJ026): Interim analysis of an open-label, randomised, multicentre, phase 3 trial. Lancet Oncol..

[67] Gridelli C., Rossi A., Ciardiello F., De Marinis F., Crinò L., Morabito A., Morgillo F., Montanino A., Daniele G., Piccirillo M.C. (2016). BEVERLY: Rationale and Design of a Randomized Open-Label Phase III Trial Comparing Bevacizumab Plus Erlotinib Versus Erlotinib Alone as First-Line Treatment of Patients With EGFR-Mutated Advanced Nonsquamous Non–Small-Cell Lung Cancer. Clin. Lung Cancer.

[68] Hiranuma O., Uchino J., Yamada T., Chihara Y., Tamiya N., Kaneko Y., Yoshimura K., Takayama K. (2019). Rationale and Design of a Phase II Trial of Osimertinib Combined With Bevacizumab in Patients With Untreated Epidermal Growth Factor Receptor-mutated Non–small-cell Lung Cancer and Malignant Pleural and/or Pericardial Effusion (SPIRAL II Study). Clin. Lung Cancer.

[69] Nakagawa K., Garon E.B., Seto T., Nishio M., Aix S.P., Chiu C.-H., Park K., Novello S., Nadal E., Imamura F. (2019). RELAY: A multinational, double-blind, randomized Phase 3 study of erlotinib (ERL) in combination with ramucirumab (RAM) or placebo (PL) in previously untreated patients with epidermal growth factor receptor mutation-positive (EGFRm) metastatic non-small cell lung cancer (NSCLC). J. Clin. Oncol..

[70] Yang J.C.-H., Shepherd F.A., Kim D.-W., Lee G.-W., Lee J.S., Chang G.-C., Lee S.S., Wei Y.-F., Lee Y.G., Laus G. (2019). Osimertinib Plus Durvalumab versus Osimertinib Monotherapy in EGFR T790M–Positive NSCLC following Previous EGFR TKI Therapy: CAURAL Brief Report. J. Thorac. Oncol..

[71] Dive C., Brady G. (2017). SnapShot: Circulating Tumor Cells. Cell.

[72] Pailler E., Faugeroux V., Oulhen M., Catelain C., Farace F. (2017). Routine clinical use of circulating tumor cells for diagnosis of mutations and chromosomal rearrangements in non-small cell lung cancer—Ready for prime-time?. Transl. Lung Cancer Res..

[73] Kapeleris J., Kulasinghe A., Warkiani M.E., Vela I., Kenny L., O’Byrne K., Punyadeera C. (2018). The Prognostic Role of Circulating Tumor Cells (CTCs) in Lung Cancer. Front. Oncol..

[74] Kulasinghe A., Kapeleris J., Kimberley R., Mattarollo S.R., Thompson E.W., Thiery J.-P., Kenny L., O’Byrne K., Punyadeera C. (2018). The prognostic significance of circulating tumor cells in head and neck and non-small-cell lung cancer. Cancer Med..

[75] Kulasinghe A., Punyadeera C., Kapeleris J., Cooper C., Warkiani M.E., O’Byrne K., Punyadeera C. (2019). Phenotypic Characterization of Circulating Lung Cancer Cells for Clinically Actionable Targets. Cancers.

[76] Pailler E., Faugeroux V., Oulhen M., Mezquita L., Laporte M., Honore A., Lecluse Y., Queffelec P., Ngo-Camus M., Nicotra C. (2019). Acquired Resistance Mutations to ALK Inhibitors Identified by Single Circulating Tumor Cell Sequencing in ALK-Rearranged Non–Small-Cell Lung Cancer. Clin. Cancer Res..

[77] Zhang Q., Nong J., Wang J., Yan Z., Yi L., Gao X., Liu Z., Zhang H., Zhang S. (2019). Isolation of circulating tumor cells and detection of EGFR mutations in patients with non-small-cell lung cancer. Oncol. Lett..

[78] Kulasinghe A., Lim Y., Kapeleris J., Warkiani M.E., O’Byrne K., Punyadeera C. (2020). The Use of Three-Dimensional DNA Fluorescent In Situ Hybridization (3D DNA FISH) for the Detection of Anaplastic Lymphoma Kinase (ALK) in Non-Small Cell Lung Cancer (NSCLC) Circulating Tumor Cells. Cells.

[79] Gkountela S., Castro-Giner F., Szczerba B.M., Vetter M., Landin J., Scherrer R., Krol I., Scheidmann M.C., Beisel C., Stirnimann C.U. (2019). Circulating Tumor Cell Clustering Shapes DNA Methylation to Enable Metastasis Seeding. Cell.

[80] Szczerba B.M., Castro-Giner F., Vetter M., Krol I., Gkountela S., Landin J., Scheidmann M.C., Donato C., Scherrer R., Singer J. (2019). Neutrophils escort circulating tumour cells to enable cell cycle progression. Nature.

[81] Aceto N., Bardia A., Miyamoto D.T., Donaldson M.C., Wittner B.S., Spencer J.A., Yu M., Pely A., Engstrom A., Zhu H. (2014). Circulating Tumor Cell Clusters Are Oligoclonal Precursors of Breast Cancer Metastasis. Cell.

[82] Au S.H., Storey B.D., Moore J.C., Tang Q., Chen Y.-L., Javaid S., Sarioglu A.F., Sullivan R., Madden M.W., O’Keefe R. (2016). Clusters of circulating tumor cells traverse capillary-sized vessels. Proc. Natl. Acad. Sci. USA.

[83] Chen K., Zhang J., Guan T., Yang F., Lou F., Chen W., Zhao M., Zhang J., Chen S., Wang J. (2017). Comparison of plasma to tissue DNA mutations in surgical patients with non–small cell lung cancer. J. Thorac. Cardiovasc. Surg..

[84] Passiglia F., Rizzo S., Di Maio M., Galvano A., Badalamenti G., Listi A., Gulotta L., Castiglia M., Fulfaro F., Bazan V. (2018). The diagnostic accuracy of circulating tumor DNA for the detection of EGFR-T790M mutation in NSCLC: A systematic review and meta-analysis. Sci. Rep..

[85] Chae Y.K., Oh M.S. (2019). Detection of Minimal Residual Disease Using ctDNA in Lung Cancer: Current Evidence and Future Directions. J. Thorac. Oncol..

[86] Abbosh C., Birkbak N.J., Wilson G.A., Jamal-Hanjani M., Constantin T., Salari R., Quesne J.L., Moore D.A., Veeriah S., Rosenthal R. (2017). Phylogenetic ctDNA analysis depicts early-stage lung cancer evolution. Nature.

